# School-Based Multicomponent Intervention to Promote Physical Activity and Reduce Sedentary Time of Disadvantaged Children Aged 6-10 Years: Protocol for a Randomized Controlled Trial

**DOI:** 10.2196/17815

**Published:** 2020-09-23

**Authors:** Caroline Maite Marie Bernal, Lena Lhuisset, Nicolas Fabre, Julien Bois

**Affiliations:** 1 Universite de Pau & des Pays de l’Adour, e2s UPPA, MEPS Tarbes France

**Keywords:** children, school, intervention, promotion, physical activity, sedentary time, attention, academic achievement

## Abstract

**Background:**

In our modern society, physical activity (PA) is decreasing and sedentary time (ST) is increasing, especially for children from disadvantaged neighborhoods. School-based interventions to promote PA and decrease ST are therefore required among this population in order to change children’s lifestyle habits. Moreover, attentional capacities and academic achievement can be enhanced by chronic PA during childhood. The relationships between these variables have been poorly studied with this population.

**Objective:**

The objective of this study is to present the rationale and methods for a randomized controlled trial among 6-10-year-old children with low socioeconomic status that will (1) evaluate the effectiveness of a school-based intervention designed to promote PA and reduce ST and (2) study the relationships between PA, ST, motor skills, attentional capacities, and academic achievement.

**Methods:**

A randomized controlled trial was conducted in 2 eligible primary schools. During academic year 2016-2017, 1 school was randomly assigned as the experiment one and the other was assigned as the control one. Five assessments times were used: baseline (T1 [November 2016] to T2 [June 2017]), follow-up (T3 [November 2017] to T4 [June 2018]), and final assessment (T5 [June 2019]). The school-based intervention included various components on different levels of the socioecological model: (1) curriculum-based program for children; (2) sensitization workshops and newsletters for parents; (3) training workshops for teachers; (4) environmental adaptation of playgrounds and reorganization of recess time; (5) time adaptation of lunch breaks; and (6) collaboration with political groups. PA, ST, motor skills, and attentional capacities were evaluated and academic achievement was recorded.

**Results:**

The presented intervention and its different assessments have been successfully implemented. In order to achieve the 2 objectives of this randomized controlled trial, data analyses are about to be completed.

**Conclusions:**

The implementation of this randomized controlled trial can help to determine effective strategies to promote PA in the context of increasing prevalence of physical inactivity among children with sedentary lifestyle which will be useful for researchers, stakeholders, and public policy makers.

**Trial Registration:**

ClinicalTrials.gov NCT03983447; https://clinicaltrials.gov/ct2/show/NCT03983447

**International Registered Report Identifier (IRRID):**

RR1-10.2196/17815

## Introduction

### Background

Physical inactivity has been recognized in the last decade as a major cause of noncommunicable disease, being held responsible for more than 5.3 million of the 57 million deaths that occurred worldwide in 2008, and for a decrease in life expectancy [1]. Furthermore, physical activity (PA) has a protective effect against more than 20 chronic diseases, including obesity [2]. Thus, the World Health Organization (WHO) has set a global action plan to increase PA levels and decrease sedentary time (ST), with the aim of “a 15% relative reduction in the global prevalence of physical inactivity in adults and in adolescents by 2030” [3]. To reach that goal, primary school children are an important target because behaviors adopted in childhood affect health habits and lifestyle choices in adulthood [4,5]. International guidelines for 6-11-year-old children recommend at least 60 minutes of moderate-to-vigorous PA (MVPA) per day as well as a reduction of sedentary behavior [3]. However, data suggest that when conservative cut points are used to define MVPA, less than half of the children meet the 60-minute MVPA recommendation [6,7]. As an example, the IDEFICS study reported an average MVPA of 49 minutes for boys and 36 minutes for girls and an average ST of 370 minutes in 8-year-old children from 8 European countries [8]. Furthermore, children of low socioeconomic status seem to have lower PA levels as well as higher ST [9]. Thus, they should be specific targets for intervention because the WHO defines the reduction of inequalities as one of its Sustainable Development Goals [3].

Many studies have investigated the effectiveness of intervention programs for children to promote PA [10-12]. Given the fact that children spend a large part of the day at school, most of the interventions are carried out in this context [11-13]. This environment, dedicated to learning, allows the use of several actions and makes it possible to include children of any social class. Some interventions increase the frequency and duration of physical education classes and include health education workshops [14,15]. These workshops usually contain information on PA and nutrition. The results of these interventions are inconclusive because the children practice less PA outside of these extra physical education classes, leading to a reduced effectiveness [16,17]. In fact, school is not the only place for children to be physically active. The socioecological model of Sallis applied to health behavior identifies different levels of factors that influence children’s behavior, ranging from personal, interindividual (family or friends), community and environmental, and societal levels [18,19]. Some interventions act only on environmental factors, such as a new playground design adapted to PA practice. This type of environmental adaptation increased light PA and decreased ST, but did not affect MVPA [20]. Thus, as underlined recently by the WHO, a system-based approach should be favored, which means that interventions have to be integrated and multilevel to actually increase levels of PA and decrease ST [3]. In fact, there is strong evidence for the efficiency of school-based interventions with family and community involvement and multilevel interventions [16]. Thus, if actions are implemented at schools, workshops for parents and teachers should be added, especially in disadvantaged neighborhood that include many children from low socioeconomic status families [16,17].

As stated by the WHO, if PA has to be increased, ST also has to be reduced, and healthy PA habits should be learned during childhood [3-5]. In fact, if 7-year-old children spend about half of their waking time in sedentary behavior, this proportion increases up to around 75% at 15 years of age [21]. Furthermore, the effects of physical inactivity and sedentary lifestyle are independent of health, especially at the cardiometabolic level [22]. Thus, specific interventions to reduce ST are needed. Usually, these interventions focus on the development of classroom material. A literature review analyzed 13 studies and showed that height-adjustable desks decreased sitting time in the classroom [23]. Active classroom lessons are also used in several intervention programs to promote PA and break down ST, leading to improvement in on-task behavior during academic instruction [24]. In order to show evidence of the hypothesized change of behavior, an objective method of measurement of PA and ST, such as accelerometry, in preintervention and postintervention periods is necessary because self-reported ST is highly underestimated [25].

To help convince the educational authorities as well as the teachers to implement such interventions, recent evidence underlines the role of physical movement in the establishment of fundamental mental processes during childhood and adolescence, leading to cognitive benefits [26,27]. PA seems to have positive effects on the attentional capacities of children as well as academic achievement [28]. In fact, physical condition would be a mediatory element between PA and executive functions that include attentional capacities [29]. In addition, the development of gross and fine motor skills has a positive impact on cognitive capacities [30]. Thus, it is necessary to explore the relationships between PA, motors skills, attentional capacities, and academic achievement for children with low socioeconomic status.

### Aims

This article aims to present the rationale and methods for a randomized controlled trial among 6-10-year-old children with low socioeconomic status that will (1) evaluate the effectiveness of a school-based intervention designed to promote PA and reduce ST and (2) study the relationships between PA, ST, motor skills, attentional capacities, and academic achievement.

## Methods

### Context

In 2016, a Pyrenean cross-cultural structure called Centre for the Promotion of Physical Activity and Health (CAPAS-City) was created to promote PA in 2 cities (Huesca [Spain] and Tarbes [France]). It involves 4 partners: the city councils of Huesca (Spain) and Tarbes (France), and 2 research groups from the University of Zaragoza (Spain) and the University of Pau and Pays de l’Adour (France), respectively. CAPAS-City was funded by the European Regional Development Fund (FEDER) programme for territorial cooperation and sustainable development of cross-border regions (Spain, France, and Andorra in this case) called POCTEFA 2014-2020 (EFA095/15). This center is in charge of developing PA programs and promotional activities that have a beneficial impact on health especially in disadvantaged populations that are more prone to health issues. It is within this framework that this study has been conducted because it is focused on children from disadvantaged neighborhoods.

### Study Design and Population

The study was designed as a randomized controlled trial with 2 arms. In a mid-sized city in the southern part of France, 2 primary schools located in the city’s disadvantaged neighborhood were invited to participate in the study. In France, neighborhoods are classified as disadvantaged on the criterion of income per inhabitant: this income is compared with the average income of the city and that of France. This defines the unfavorable social and economic conditions of the neighborhood and its inhabitants. For example, in the neighborhood considered in this study, the unemployment rate is the highest in the city (26% vs 18%) as well as the prevalence of social housing (49% vs 23%).

**Figure 1:**
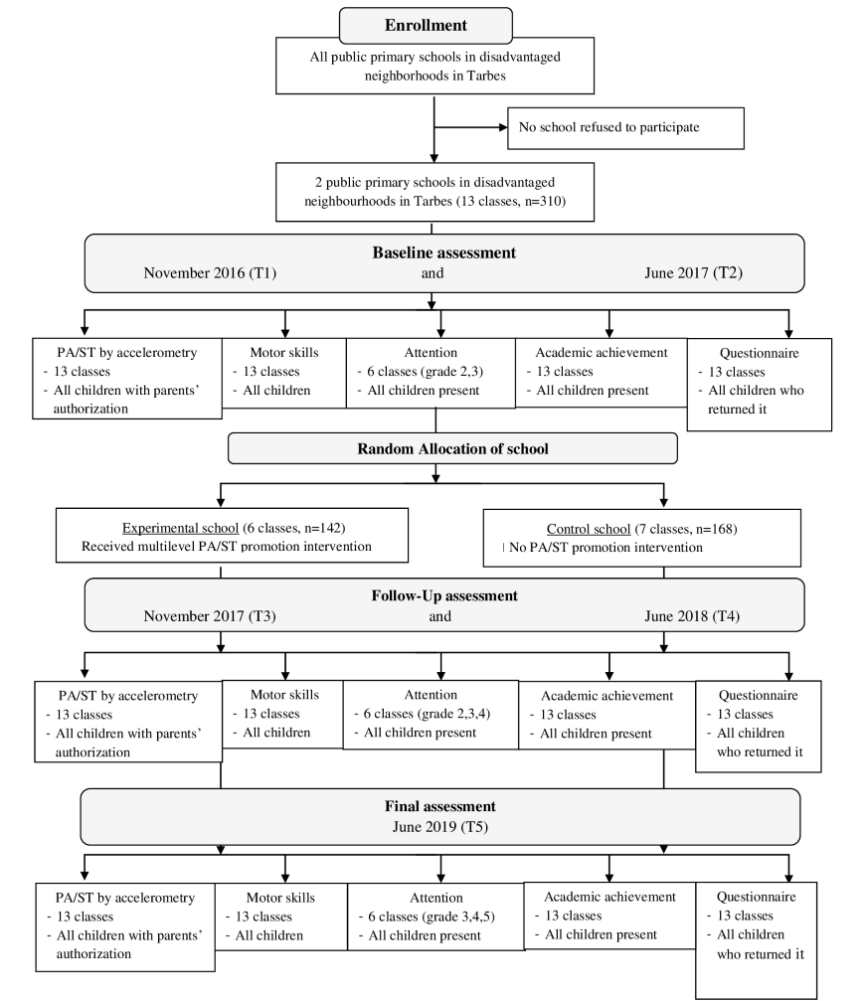
Flowchart of participants. PA: physical activity; ST: sedentary time.

Both primary schools agreed to participate in the project. They included children from grade 1 (6 years old) to grade 5 (10 years old) and had never benefited from any intervention in the field of PA. Schools were randomly assigned to either experimental (School A) or control group (School B; see Figure 1). Because the intervention is school based, all the children were included in the study (n=141 for school A and n=148 for school B). Based on studies already published on the same theme, the effects of the intervention are expected to be moderate (standard difference≈0.32, equivalent to an average change in MVPA measurement of 8 minutes per day; SD 18 minutes). In addition, in order to detect a difference of 0.32 with a statistical power of 0.8, a significance level of .05, and a retention rate of approximately 91% with a pre/post comparison, the number of participants should be 70. As a precaution, more children were included. Thus, the number of children enrolled was above the minimum required.

During the academic year 2016/2017, the following baseline assessments were carried out in these 2 schools: measurements of PA and ST, motors skills, attentional capacities, and academic achievement as well as questionnaires completed by parents. These assessments were carried out in November/December 2016 (T1) and May/June 2017 (T2). Children in both schools had to have parental permission to wear the accelerometer for PA and ST measurement. Motor skills were assessed for all the children at school and their records of academic achievement were collected. The measurements of attentional capacities involved only children in grades 2 and 3.

During the academic school year 2017/2018, the experimental school benefited from a school-based multilevel intervention to increase PA, reduce ST, and make children, parents and teachers aware of the importance of PA for health. This intervention program involved all the children from grade 1 to grade 5. During this interventional year, the same periods of assessment as those carried out at baseline were repeated in November/December 2017 (T3) and May/June 2018 (T4). A final assessment was carried out in June 2019 (T5) after 1 year without any special intervention in the experimental school.

### Baseline and Follow-Up Assessments

Table 1 reports the number and percentage of children who participated in the assessment times (T1–T2–T3–T4–T5) in the experimental school (School A) and in the control school (School B).

**Table 1:** Children’s participation in baseline, follow-up, and final assessments in the 2 schools.

Measurements			T1	T2	T3	T4	T5
**School A**							
		Accelerometry, n/N (%)	91/142 (64.1)	96/142 (67.6)	74/135 (54.8)	45/137 (32.8)	71/134 (53.0)
		Attention, n/N (%)	48/142 (33.8)	49/142 (34.5)	44/135 (32.6)	47/137 (34.3)	53/134 (39.5)
		Motor Skills, n/N (%)	130/142 (91.5)	129/142 (90.8)	125/135 (92.6)	110/137 (80.3)	109/134 (81.3)
		Academic Achievement, n/N (%)	118/142 (83.1)	118/142 (83.1)	105/135 (77.8)	105/137 (76.6)	95/134 (70.9)
		Questionnaire, n/N (%)	90/142 (63.4)	90/142 (63.4)	60/135 (44.4)	60/137 (43.8)	58/134 (43.3)
**School B**							
	Accelerometry, n/N (%)		121/168 (72.0)	116/168 (69.0)	90/154 (58.4)	51/154 (33.1)	86/175 (49.1)
	Attention, n/N (%)		65/168 (38.7)	66/168 (39.3)	58/154 (37.7)	56/154 (36.4)	71/175 (40.6)
	Motor Skills, n/N (%)		152/168 (90.5)	153/168 (91.1)	153/154 (99.4)	153/154 (99.4)	158/175 (90.3)
	Academic Achievement, n/N (%)		156/168 (92.9)	156/168 (92.9)	140/154 (90.9)	140/154 (90.9)	99/175 (56.6)
	Questionnaire, n/N (%)		113/168 (67.3)	113/168 (67.3)	73/154 (47.4)	73/154 (47.4)	57/175 (32.6)

#### Physical Activity and Sedentary Time

GT3X Accelerometers (ActiLife) were used as a valid objective measure to assess the levels of PA and ST [31-33]. These assessments were performed in T1, T2, T3, T4, and T5. Children wore the accelerometer on the right side of the hip, adjusted with an elastic belt, from morning to evening for 8 consecutive days. They removed it to sleep, during the shower, and for aquatic activities. The first day was excluded from the analysis and the data collection was carried out over 5 weekdays and 2 weekend days. The accelerometer had to be worn for at least 10 hours on weekdays and 8 hours on weekend days to be included in the analysis, according to the optimal methodological approach for accelerometry [34]. In addition, if the accelerometer detected at least 10 minutes of activity of 0 counts (cts), allowing this quota of time 1-2 minutes between 0 and 100 cts, this time was determined as “no wear time.” To be considered as representative of children’s usual behavior, wear time had to be valid for at least 3 weekdays and 1 weekend day [35].

For the treatment of accelerometer data, ActiLife version 6.13.3 software (ActiGraph) was used. In order to measure children’s PA, which is described as spontaneous, it is preferable to use epochs of less than 15 seconds which makes it possible to detect more accurately the changes in the child’s PA intensities [36]. Thus, we used 1-second epochs and 5 cut points to establish the different intensity categories [31,37]: sitting time 0-99 cts, standing time 99-300 cts, light PA 301-2295 cts, moderate PA 2296-4011 cts, and vigorous PA 4012 cts and more. To obtain ST, the first 2 cut points were added, and thus the cut point corresponding to ST was 0-300 cts.

In order to better understand the behavioral changes, PA and ST were analyzed within specific periods of the day corresponding to the French cultural children’s schedule: time before school (08:00-08:30); morning and afternoon school time (08:30-12:00 and 14:00-16:00); lunch time (12:00-14:00); after-school time (16:00-19:00); evening time (19:00-21:00). During each of these periods, to be valid, the accelerometers had to be worn for 80% of the standard segment time, with this standard segment time being defined as the length of time in which at least 70% of the participants wore the monitor [31].

#### Motor Skills

Global motor skills were evaluated with 3 tests issued from the Eurofit Test Battery [38,39] because it is valid and reproducible for school children aged 6-18 [40]. They were implemented as described in the Eurofit Handbook [38]. Cardiorespiratory fitness was also measured with the PREFIT 20-m Shuttle Run Test, an adaptation of the original 20-m Shuttle Run Test for children [41]. These tests were carried out at T1, T2, T3, T4, and T5.

##### Standing Broad Jump

This test is included in the Eurofit Test Battery and measures the explosive power of the legs and intersegmental coordination [38].

##### Platte Tapping Test

This test is included in the Eurofit Test Battery [38]. It measures the speed and coordination of the upper limb.

##### 6 × 5-m Shuttle Run

This test was adapted from the 10 × 5-m Shuttle Test of the Eurofit Test Battery [38]. It measures running speed and agility.

##### Cardiorespiratory Fitness: PREFIT 20-m Shuttle Run Test

The aerobic capacity was measured with an adapted version of the original 20-m Shuttle Run Test [42]: the PREFIT 20-m Shuttle Run Test, more appropriate for young children [41]. Children had to run back and forth between 2 lines 20 m apart with an audio signal giving the rhythm of the corresponding speed. The running speed increased by 0.5 km/h each minute. Some adaptations of the original test were made to fit children’s capacities by decreasing the initial speed (ie, 6.5 km/h instead of the original 8.5 km/h) and by having 2 evaluators running with a reduced group of children (eg, 8-12 of the same age) in order to provide an adequate pace. The test ended when the child failed to reach the end lines concurrent with the audio signal on 2 consecutive occasions or when the child stopped because of exhaustion. The results were expressed as the number of laps completed. From this value, the maximal aerobic shuttle running speed (km/h) and the maximal oxygen uptake (VO_2max_ mL/kg/min) can be calculated.

#### Attentional Capacities

The computer-based modified Erickson Flanker Task was used as a variant version of the Flanker-Task [43,44]. It was designed with the software SuperLab 4.5 (Cedrus Corporation). It permits the measurement of inhibition and cognitive flexibility, which are identified as attentional capacities [45]. Inhibition refers to focusing on essential elements of the environment while not focusing on disturbing elements, and cognitive flexibility involves changing from one cognitive operation to another. The sustained attention was evaluated as well. The task was to respond to a target stimulus while ignoring distracting stimuli presented on a 15-in. computer screen. In this version the stimuli were pictograms of a fish oriented either toward the left or toward the right. The child was instructed to press on the “P” key of the keyboard (located on the right hand side) with the right hand for target fishes facing to the right, and on the “A” key of the keyboard (located on the left hand side) with the left hand for target fishes facing to the left. The test included 3 conditions: (1) standard flanker, (2) reverse flanker, and (3) mixed condition. In the standard flanker condition, the fishes were blue. The target stimulus was the fish located in the middle of the screen. It could be oriented to the left or to the right and could be accompanied on either side by other fishes facing either direction and they had to be ignored (ie, distractors). In the reverse flanker condition, the fishes were pink. In contrast to the previous condition, the target stimuli were the fishes located on either side of the screen while the middle fish had to be ignored (ie, the distractor). These 2 conditions require sustained attention to remember the initial instruction and stay focused throughout the test, and inhibition of the appropriate distractor. In the mixed condition, trials from the standard condition (with blue fishes) and trials from the reverse condition (with pink fishes) were randomly mixed up. The child had to adapt to the variation of the rule to be applied. Thus, this condition required cognitive flexibility on top of sustained attention and inhibition.

For each condition, after being instructed in the task, the child practiced with 2 familiarization blocks of 7 stimuli: 1 block with feedback on the correctness of his/her answer and 1 block without feedback. If necessary, these blocks were repeated until it was made sure that the task was clearly understood. The child was asked to respond as quickly and as accurately as possible by pressing on the appropriate key (“P” or “A”) according to the stimulus [44].

The reaction time and the correctness of the answers were recorded to measure sustained attention, inhibition, and flexibility. This test was carried out at each measurement period (T1, T2, T3, T4, and T5), only for children in grades 2, 3, and 4. Children were assessed during school morning from 8:30 to 12:00. The measurements took place in a separate classroom and 1-4 children were assessed at the same time. Each child was placed in a corner of the classroom in front of a 15-in. screen, with the attendance of 1 specialist researcher. The instructions were given verbally and individually by the specialist.

#### Academic Achievement

Measures of academic achievement for each child and for both schools were collected at the beginning of each academic year, that is, at baseline (T1), follow-up assessment (T3), and final assessment (T5). Criteria of assessment of the academic achievement were the same for both schools. These data were provided by the local educational services. Percentages of success for “reading,” “spelling,” “arithmetic,” and “mathematics” were collected.

#### Questionnaire

In addition to collecting academic achievement data, a questionnaire was distributed at the beginning of each school academic year (ie, T1, T3, and T5). Parents were asked to complete a questionnaire in order to obtain (1) sociodemographic information (age and gender of the child, socioeconomic data measured by the 4-item scale “Family Affluence Scale II” [FAS II] [46], marital status, and level of qualification of the parents) and (2) subjective information related to children’s PA and ST behavior (number of sports and outdoor activities practiced, parental perception of the competence of their children in PA practice, sedentary behavior of the child [time spent playing video games, watching television, and artistic and musical activities]).

### Statistical Analysis

STATISTICA version 12.5 (StatSoft/Dell) for Windows and R software will be used. Descriptive statistics of MVPA and ST for the whole day and within the different specific periods of the day will be calculated for both schools at the different assessment times. To examine the effectiveness of the intervention to promote PA and reduce ST, ANOVAs contrasting 2 schools × 5 assessment times with repeated measurement on the last factor will be used on MVPA and ST. Furthermore, chi-square analysis will be carried out to compare the number of children between experimental and control groups who comply with MVPA recommendations for the overall day and during school time.

To explore the longitudinal relationship between PA, motor skills, attention, and academic achievement, multiple regression analyses will be conducted to determine which variables will predict academic achievement scores [47]. We will attempt to examine whether PA or motor variables predict academic achievement through attention. As there are many variables in each category, a principal component analysis will be used to select the variables that are most correlated. Finally, the decision tree process will be used to examine more accurately the longitudinal relationships between the variables selected.

### Intervention Program

Because interventions that are integrated and multileveled seem to be more effective in triggering behavioral changes concerning the levels of PA and ST, this program has been conceived as a school-based approach with interventions at different levels of factors that influence children’s behaviors [3,18,19]. More specifically, it is directed to the children (intrapersonal level), but also to their teachers and parents (interindividual level) and to their environment (physical and organizational levels). Furthermore, to be integrated, it has to be adapted to the real needs of the particular population to which it is dedicated. Thus, PA and ST data from the baseline assessment were analyzed to tune and adapt the intervention program.

#### Principal Outcomes From Measurements at Baseline Concerning PA and ST

On average, children aged 8-10 spent 67.20 (SD 17.83) minutes in MVPA and 602.97 (SD 36.32) minutes in ST on weekdays. At different periods of the school day, it appeared that (1) during school time, the international recommendation to spend 30 minutes in MVPA were fulfilled by only 26.92% of the grade 2 children and 5.40% of the grade 5 children. In fact, children spent 22-27 minutes in MVPA during school time while they were sedentary for 260-270 minutes. Thus, it was confirmed that school is a highly sedentary place where PA has to be promoted during recess and also during class time through sedentary breaks and active learning strategies. (2) During lunch time, sedentary activities represented 80% of the period (ie, 88-93 minutes of ST against 10-13 minutes of MVPA), suggesting that this period of the school day could be used for children to be more active and less sedentary through an organizational process. (3) Before school, only 2-3 minutes were spent in MVPA while 22 minutes were ST, suggesting that active transportation to school was scarce or that school is very close to home for some children. Thus, children’s and parents’ sensitization to active transportation should be developed.

#### PA and ST Intervention Program

According to the socioecological model and to the analyses of PA and ST at baseline, specific intervention actions have been carried out at the different levels of the model. Their content and objectives are detailed in Table 2.

The timings of these actions were coordinated across the different levels of the model. Thus, the contents of the different workshops for the children and the parents were coordinated with the training for the teachers. For example, workshops 1-3 for children took place at the same time of year as sensitization workshops 1 and 2 for parents and training workshop 1 for teachers in order to allow children to discuss and exchange views on these subjects (“what is PA?”, “what is ST?”) with the adults around them and for the teachers to reactivate part of the knowledge when they had the opportunity. Then, feedback from the baseline assessment of PA and ST levels and motor skills was given to the children, parents, and teachers. This was coordinated with workshops 5 and 6 for the children. This led to actions, learning, and discussions with the different actors (ie, children, parents, and teachers) in order to increase PA and decrease ST. Thus, around that time of year, children explored and tested concrete “activities in the schoolyard,” parents came to sensitization workshop 3 (How can I influence my child’s PA?), and teachers were trained in active classroom and sedentary breaks (training workshop 2). This training was followed by a demonstration of these activities in the class (Active classroom and sedentary breaks). Furthermore, during training workshops 3 and 4, teachers discussed strategies to increase children’s PA and the different factors that can influence it. In parallel, some environmental adaptations were carried out. As noted earlier, the baseline assessment led the research team to realize that lunch time was highly sedentary (80% [~95 minutes] of the 120 minutes lunch) when it should be devoted to some active leisure. Therefore, discussions were held with the different staff working in the canteen to reorganize this time so that children spent less time sitting and more time playing and moving (Organizational modification of lunch break). In parallel, a discussion was carried out with the teachers to optimize the organization of the different areas of the schoolyard based on the games to allow children to find their favorite activity and then be more active (Organizational modification of recess games).

Around the middle of the school year, delineations of games in the schoolyard were created by the education department of the town hall (physical and material modifications of the schoolyard), following a participatory bottom-up approach. In fact, the first ideas came from the children (workshop 4: each child drew what he wanted to have in his schoolyard) and then a first selection was done at the class level (design of the schoolyard from the different individual suggestions). Finally, a consultation between the teachers, the research team, and the representatives of the town hall led to the final choices. Furthermore, discussions were carried out on the role of the surrounding environment in facilitating or preventing PA practice at the children’s level with the “photovoice workshop,” as well as at parents’ level (sensitization workshop 4) and at teachers’ level (training workshop 4).

To conclude the intervention, assessments were made with children (workshop 7), parents (sensitization workshop 5), and teachers (training workshop 5) to discuss the behavioral changes that took place and the strategies that were put into place (what worked well or what didn’t?). A drawing contest was organized among children (workshop 8): they were asked to draw themselves in an active situation, and their drawings were presented to the education community and the parents at the end of the school year.

Finally, different political levels were involved during the intervention, such as the different services of the city town hall and the French national education system, which allowed the research team to conduct its actions.

**Table 2:** The different axes of the intervention: content and objectives.

Theme of each action according to the level of the socioecological level				Content	
**Intraindividual (level 1): children**					
		Workshop 1: “What is PA?”		Information about PA was given: the different intensities, the principal differences between PA and sport, and the benefits of PA on health.	
		Workshop 2: “How to practice PA?”		Different activities were presented to the child to be more active at school, in his/her neighborhood, in a sports club, and at different moments of the day.	
		Workshop 3: “What are sedentary habits?”		Information about the different sedentary behaviors that children can have in a day was given, as well as the effect of cumulative ST on health. The possibilities to decrease ST at each time of the day were analyzed.	
		Workshop 4: “What would you like to draw on the schoolyard to play, run, and have fun?”		Children had to draw schoolyard equipment and materials that they would like to have, to allow them to have fun and to move around. These proposals were then studied in each class, classified, and selected to make a proposal per class.	
		Workshop 5: “How does the accelerometer measure PA and ST?”		The mechanical operation of the accelerometer was studied. The data from the accelerometer were analyzed. The children determined their compliance with the WHO guidelines.	
		Workshop 6: “Do I practice enough PA every day to be healthy?”		An analysis of the time devoted to the practice of PA and ST every day was made for each child. Discussions were held with the children about these data and strategies to further increase PA and decrease ST. They identified and worked on barriers to PA practice.	
		Workshop 7: “Have I changed my PA practice? Am I trying to be less sedentary?” and “What did I learn about PA and ST?”		An assessment was made with children on behavioral changes in terms of PA and ST and on the knowledge acquired on PA, ST, and more generally on health.	
		Workshop 8: Drawing contest		A drawing contest on the theme “I’m moving” was done at the end of the intervention period.	
		Activities in the schoolyard: “What games can I play...?”		A session was organized in the schoolyard to identify games that can be played in the schoolyard or in leisure time, alone or with friends. For example: “what games can I play with a cord?” The children imagined and listed games and demonstrated them.	
		Active classroom: “Spelling and mathematics”		Demonstration of active classroom workshops for further use by the teachers: “Spelling activity,” in which children had to touch different parts of the body while spelling out the letters of words. The words were more or less complicated depending on the grade of the children. (2) “Arithmetic exercise,” in which children had to make a jump after having said the result of the addition or multiplication.	
		Sedentary breaks: “Relaxation and breathing”		The activity break included breathing, relaxation, and visualization exercises, or body movements such as motor coordination, balance, and flexibility exercises. These sedentary breaks did not include academic knowledge.	
		Activities outside of school: Photovoice workshop		Excursion to the school neighborhood to observe the areas in which one can practice PA and to identify the areas dangerous for practice. The children took pictures of the different areas with tablets. Back in class, the children showed their photos to their classmates and explained why they took this picture (if it was possible to practice PA in this area, or if it was a dangerous area).	
		Feedback		Individual summary sheets with PA and ST levels and performance at the different motor tests were given to each child in a graphic format after each assessment. Explanations and consideration took place in the classroom.	
**Interpersonal (level 2): parents**					
		**Information meeting**		The overall project was presented (ie, all the activities planned for parents and children and teachers, school, and the involvement of local political groups).	
			Workshop 1: “What is PA?” and “How to practice PA?”	Information about PA was given. Consideration of the different opportunities to practice PA: for the parents themselves and with their children. Facilitators and barriers to PA practice were identified.	
			Workshop 2: “What is ST?”	Information about ST was given: different sedentary behaviors they can have in a day and the effect of cumulative ST on health. This included the study of different possibilities to decrease ST at each time of the day for them and their child.	
			Workshop 3: “How can I influence my child’s PA? How do my child’s friends influence him/her in his/her practice of PA and health behaviors?”	The influence of parents and friends on the health behaviors of the child was studied.	
			Workshop 4: “Is the environment adapted to practice physical activity?”	The different pictures taken by the children in the “Photovoice Workshop” were analyzed and discussed. A special point was made about the facilitators and the barriers to practice of PA, for them and for their children.	
			Workshop 5: “Have I changed my PA practice? Am I trying to be less sedentary? Is this also the case for my child? What did I learn about PA and ST?”	An assessment was made of their own behavioral changes in terms of PA and ST as well as for their child and of the knowledge acquired with these workshops on PA, ST, and more generally on health.	
		Newsletter		After each workshop, all parents received a newsletter with the principal information given during the workshop.	
		Feedback		Individual summary sheets with PA and ST levels and performance at the different motor tests were given to each child in a graphic format after each assessment. Parents had access to these.	
**Interpersonal (level 2): teachers**					
	Workshop 1: “What is PA? What is ST and sedentary habits?”			Theoretical knowledge about PA and ST was proposed.	
	Workshop 2: Strategic issues to increase children’s PA			Consideration of how to include information related to PA in classes: how to increase PA and decrease ST at school and especially in the classroom.	
	Workshop 3: Concrete formation on active classroom and sedentary break activities			Ideas on how to conduct an active classroom were presented: (1) proposal of active classroom exercises and sedentary breaks (breathing, relaxation, and visualization exercises, or body movements such as motor coordination, balance, and flexibility exercises); (2) advice and guidance on the implementation; (3) material organization of the classroom to increase movement, etc.	
	Workshop 4: Main influences around the children			A discussion was held on the influence of parents, friends, and teachers on children’s health behaviors. Then the environmental factors facilitating, or not, the practice PA were discussed. This led to the construction of a multilevel model and the presentation of the principal theories used in this study (ie, the socioecological model and the self-determination theory).	
	Workshop 5: Final assessment of the program intervention			An assessment of the school-based actions they had implemented was made: a discussion on their opinion about the intervention, its strength and weaknesses, and its effects on their PA and ST behaviors as well as on the children’s and their parents.	
	Feedback			The general PA and ST levels were presented to the teachers after each assessment.	
**Environmental (level 3): school**					
	Physical and material modifications of the schoolyard			After children’s workshop 4, each class made a design proposal for the schoolyard (marks to be drawn and materials to add for recess). An assessment of these proposals was made with the teachers, the research team, and the representatives of the education department of the town hall, and a choice was made about the implementation of the material required. It led to delineation of different games in the different schoolyards (ie, football field, squares with numbers and letters, snail hopscotch) and acquisition of small material (ie, balls, ropes).	
	Organizational modifications of lunch break			In line with the baseline measurement analyses, actions to reduce ST during lunch break were engaged in cooperation with the canteen’s agents and services. The aim was to move from 90 minutes of sitting time to 50-60 minutes in order to free up time to play in the schoolyard before going back to the classroom. Thus, the research team proposed changes to the organization (ie, 2 canteen services, table-based group organization) from which the educators in charge of lunch time made a choice.	
	Organizational modifications of recess games			The schoolyard was divided into different areas dedicated to specific games during recess and lunch time. This provided an opportunity to have a diversity of games and sports to play together or alone (instead of having one game/sport taking all the schoolyard). The schedule was proposed by the children and supervised by the teachers.	
**Political (level 4): local politic groups**					
		Collaboration with the city town hall		The city town hall helped in the implementation of the intervention. It allowed the interactions with the canteen agents for the reorganization of the lunch time and the drawings in the schoolyard were made by the education service of the city.	
		Collaboration with national education authorities		They authorized the implementation of the school-based intervention and helped with the organization of the actions. In addition, these authorities made it possible to officially validate the training for teachers as part of their professional learning curriculum.	
		Study developed by “CAPAS-City”		This study was conducted by CAPAS-City (founded by the European Regional Development Fund [FEDER]): this center is in charge of developing PA programs and promotions actions.	

### Ethics Approval and Consent to Participate

This study has been approved by the Comité de Protection des Personnes Sud Mediterranée III and has been registered at ClinicalTrials.gov under the identifier NCT03983447. Parents/guardians were informed of the intervention project by a written letter. This letter contained a consent form. Afterward, a public meeting with the research team was organized in each school so that parents could come to ask any questions. Then, one of the parents or guardians had to sign the consent form in order to permit the children’s participation in the study. The parents/guardians had to give this consent form to the child, who gave it to the teachers. The researchers collected the consent forms either directly after the meeting or later from the teachers.

### Availability of Data and Materials

Data sharing is not applicable to this article as no data sets were generated or analyzed during this study.

## Results

The presented intervention and the different assessments have been successfully implemented. In order to achieve the 2 objectives of this randomized controlled trial, data analyses are about to be completed. Two articles are planned: the first one will evaluate the effectiveness of the multicomponent school-based intervention and the second one will study the relationships between PA, ST, motors skills, attentional capacities, and academic achievement.

## Discussion

The most recent literature review found a lack of evidence for the effectiveness of school-based and multileveled interventions to promote PA, despite the fact that those using the socioecological model are among the most promising [10,11,17].

Thus, this article presents an improved experimental methodology in order to (1) evaluate the effectiveness of a school-based intervention to promote PA and reduce ST and (2) examine the relationships between PA, motor skills, attentional capacity, and academic achievement among disadvantaged primary school children. It can thus contribute to providing crucial information in the field of PA promotion during childhood. First, our experimental methodology used accelerometry, which is an objective method to measure PA and ST. Second, the longitudinal aspect of our study provides a follow-up for a diversity of variables related to movement and cognition over successive years during childhood. These longitudinal measures lead to a more precise understanding of the evolution of the interventional effects from the diagnostic to the follow-up measurements. Finally, the configuration of our study makes it possible to measure potentially indirect effects of the intervention on motor skills and attentional capacities.

An important aspect of this study is that the actions implemented in this intervention are not only based on the relevant literature [10-24], but also on the principal outcomes from the baseline measurement concerning PA and ST. As a consequence, the intervention has been adapted to the context and to the specific needs, which probably contributes to its effectiveness together with the fact that it has been constructed with the different actors involved and that it addresses the different levels of the socioecological model [19]. The description of this protocol could be of use to researchers in the field of PA promotion as well as to teachers of children from disadvantaged neighborhoods, to help them design actions facilitating well-being and academic success in the relevant social climate. In the long term, the objective of this project is to carry out the school-based intervention to promote PA and decrease ST in several primary schools of this French city. The next step will be to replicate the same intervention in the school that has been assigned as a control group. The effectiveness of this next intervention will also be studied.

In conclusion, physical inactivity and sedentary behavior are major public health problems. The implementation of this randomized controlled trial can help to determine effective strategies to promote PA in the context of increasing prevalence of physical inactivity among children with sedentary lifestyles. Understanding these strategies is a real necessity for researchers, stakeholders, and public policy makers seeking to establish health promotional actions for the population.

## Appendix

Multimedia Appendix 1 CONSORT-EHEALTH checklist (V 1.6.1).

## Abbreviations

FAS II: Family Affluence Scale II
MVPA: moderate to vigorous physical activity
PA: physical activity
ST: sedentary time
WHO: World Health Organization
